# Estimation of Soil Erosion to Define the Slope Length of Newly Reconstructed Gentle-Slope Lands in Hilly Mountainous Regions

**DOI:** 10.1038/s41598-019-41405-9

**Published:** 2019-03-18

**Authors:** Zhen Han, Shouqin Zhong, Jiupai Ni, Zhonglin Shi, Chaofu Wei

**Affiliations:** 1 College of Resources and Environment, Southwest University, Chongqing, 400715 China; 2 Key Laboratory of Arable Land Conservation (Southwestern China), Ministry of Agriculture, Chongqing, 400715 China; 3 Key Laboratory of Mountain Surface Processes and Ecological Regulation, Institute of Mountain Hazards and Environment, Chinese Academy of Sciences, Chengdu, 610041 China

## Abstract

Farming plot construction engineering in hilly areas plays an important role in the mechanization, large-scale production and industrialization of agriculture. The method is undertaken to improve water and soil conservation, enhance soil fertility and extend machinery agriculture. However, the positive effects of engineering require years to mature. The properties of newly reconstructed soil are not sufficient, i.e., with poor structure and low water holding capacity, resulting in deterioration of its physical properties and erosion. To date, most studies on plot characteristics and soil properties in farming plot construction engineering have neglected the influence of soil erosion. This paper addresses soil erosion characteristics at sites to define the appropriate slope length for newly reconstructed gentle-slope lands. Six field plots with a 10° slope gradient and different lengths (5, 10, 20, 30, 40, and 50 m) were established under natural rainfall and simulated overland flow conditions. The soil detachment rate, runoff shear stress and stream power exhibited the same trends as runoff and soil loss. The soil erosion characteristics varied at sites with different slope lengths, and the degree of soil erosion reached its minimum on gentle-slope land sites of 30 or 40 m. Therefore, 30–40 m slope lengths may be the recommended range to control soil loss from newly reconstructed gentle-slope lands. The conclusions of this study provide theoretical guidance for farming plot construction engineering, which can promote the sustainable development of cultivated land resources in hilly mountainous regions.

## Introduction

Soil erosion is one of the most challenging environmental problems faced by many countries worldwide^1^. To date, many studies have examined the erosion of stabilized soils; however, the erosion of newly reconstructed soil, which exhibits unstable behavior after farming plot construction, is often ignored^2^. Farming plot construction is the process of optimizing the reorganization of land resources. The core concept is a change in the physical form of the field, which includes the merging, and leveling of fields and the construction of ridges. As the basis of modern soil engineering, farming plot construction is a man-made disturbance of natural cultivated land that has a great influence on cultivated land and the ecological environment^3,4^. Sloping farmland is an important farmland resource in hilly mountainous regions in China with intense fragmentation^5,6^. Farming plot construction engineering was introduced into rural land development to mitigate the impact of natural conditions on plowland fragmentation^7^. As a soil restoration and reconstruction or soil and water conservation project, farming plot construction not only optimizes soil recovery and reorganizes land resources^8^ but also enhances soil agglomeration and reduces land fragmentation^4^. Gentle-slope land refers to farming plots with large areas in which the terrain slope is less than 10° and the land meets the requirements of agricultural mechanization or large-scale land management^5^. Typically, the shape of the plots after completion of the project is more conducive to mechanized agricultural farming. However, mechanical disturbance results in a variety of changes in soil ecosystem functions, such as soil erodibility and water and soil nutrient cycling^9^. Changes in these soil properties may alter soil hydrological properties that are closely related to runoff occurrence and sediment transport^10,11^, thereby making the soil more susceptible to erosion^12^. Soil erosion, which is caused by the slope length^13–15^, steepness, topsoil condition, rainfall^16^, particle size distribution, and slope aspect, is one of the main causes of soil nutrient losses and soil fertility decline. Soil erosion is also an important consideration in farming plot construction^17,18^. Some researchers have reported that farming plot construction may help rehabilitate the ecological environment by decreasing soil erosion, improving the agricultural environment and enhancing the ecological quality^19^. However, three phases should be considered when evaluating the effect of farming plot construction on soil erosion: the construction, recovery and balance periods^4^. Improvement of soil erosion through engineering is increasingly obvious during the balance period^8^. Engineering transformation measures conducted during the construction period will destroy the original soil structure of the fields. Importantly, the organic matter content of the newly reconstructed soil is low during the recovery period, and a large amount of gravel or stone is found in the soils. Soil drought is severe due to the low soil water preservation capability and the high soil water evaporation rate, which can weaken the ability of the soil to resist erosion; thus, soil erosion occurs easily^20^. Therefore, erosion of newly reconstructed soil during the recovery period exhibits unstable behavior after farming plot construction that cannot be ignored.

The existing literature on the effects of farming plot construction mainly focuses on four aspects: (1) these projects weaken the diversity of agricultural landscapes^21,22^; (2) these projects may cause a series of adverse ecological environmental impacts, while increasing the effective cultivated land area and improving the soil quality^18,23^; (3) the system evaluates farmland consolidation in different regions as well as, the economic, social and ecological benefits of the project^24^; and (4) the spatial variation in soil nutrients and the characteristics of soil quality evolution are evaluated in farming plot construction projects on a microcosmic scale. In addition, relatively few studies have investigated the relationship between soil erosion and gentle-slope lands after farming plot construction^2^.

Under natural rainfall and simulated overland flow conditions, this study uses newly reconstructed soils on gentle-slope lands after farming plot construction as the research objects and investigates the runoff and sediment generation processes of six plots with different gentle-slope land values (5, 10, 20, 30, 40 and 50 m). The objectives of the study were to (1) define the seriousness of soil erosion on gentle-slope lands after farming plot construction, (2) determine the appropriate slope length to control soil loss from newly reconstructed soil and (3) provide theoretical guidance for the control and monitoring of gentle-slope lands erosion after farming plot construction in hilly mountainous region.

## Results

### Erosive precipitation events

After monitoring the farming plots during the rainy season, six erosive precipitation events were recorded by rain gauges. The characteristics of these erosive precipitation events included the rainfall capacity, duration, intensity and hourly maximum rainfall intensity. The results are shown in Table 1.

**Table 1:** Rainfall characteristics of six erosive precipitation events.

Rainfall date	Rainfall capacity (mm)	Rainfall duration (h)	Average rainfall intensity (mm h^−1^)	Peak rainfall intensity per hour (mm h^−1^)
2017.6.2	36.5	7.16	5.1	12.5
2017.6.15	58.0	17.06	3.4	4.5
2017.6.29	81.8	19.48	4.2	5.5
2017.7.3	45.3	12.58	3.6	5.5
2017.8.28	74.0	22.43	3.3	5.0
2017.8.30	32.8	3.90	8.4	12.5

**Figure 1:**
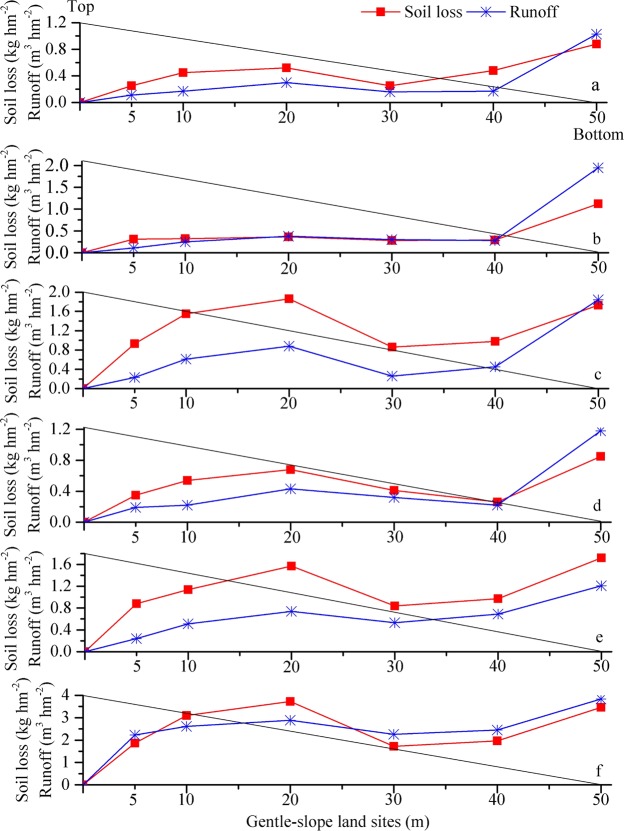
Soil loss and runoff at sites with different slope lengths (5, 10, 20, 30, 40 and 50 m) along gentle-slope lands under erosive precipitation events with diverse rainfall capacities (**a**-36.5 mm; **b**-58.0 mm; **c**-81.8 mm; **d**-45.3 mm; **e**-74.0 mm; and **f**-32.8 mm). The oblique line in the figure represents the surface of the gentle-slope land.

The soil loss and runoff characteristics for sites with different slope lengths (5, 10, 20, 30, 40 and 50 m) along the gentle-slope lands under erosive precipitation events with diverse rainfall capacities are shown in Fig. 1. The ANOVA and T-test showed that the slope length had a significant effect on runoff and soil loss (*p* < 0.01). When the rainfall capacity and average intensity were both low, the amounts of soil loss and runoff were correspondingly reduced. Although the rainfall capacity of 32.8 mm shown in Fig. 1f was small, the soil loss and runoff were high due to the larger average rainfall intensity. Fig. 1 also showed that the change trends of soil loss and runoff with slope length were similar, with an initial increase, a subsequent decrease, and a final increase. Certain differences in soil loss and runoff were observed for the different gentle-slope land sites. Among them, the soil loss and runoff due to rainfall events shown in Fig. 1a–c,e,f reached their minimum values on the 30 m gentle-slope land site, whereas the results shown in Fig. 1d reached their maximum values at the 40 m site. In these six rainfall events, the 20 m gentle-slope land site had increased soil loss and runoff; these measures reached a minimum at 30 or 40 m, and an increasing trend was observed at 50 m.

### Simulated overland flow

**Figure 2:**
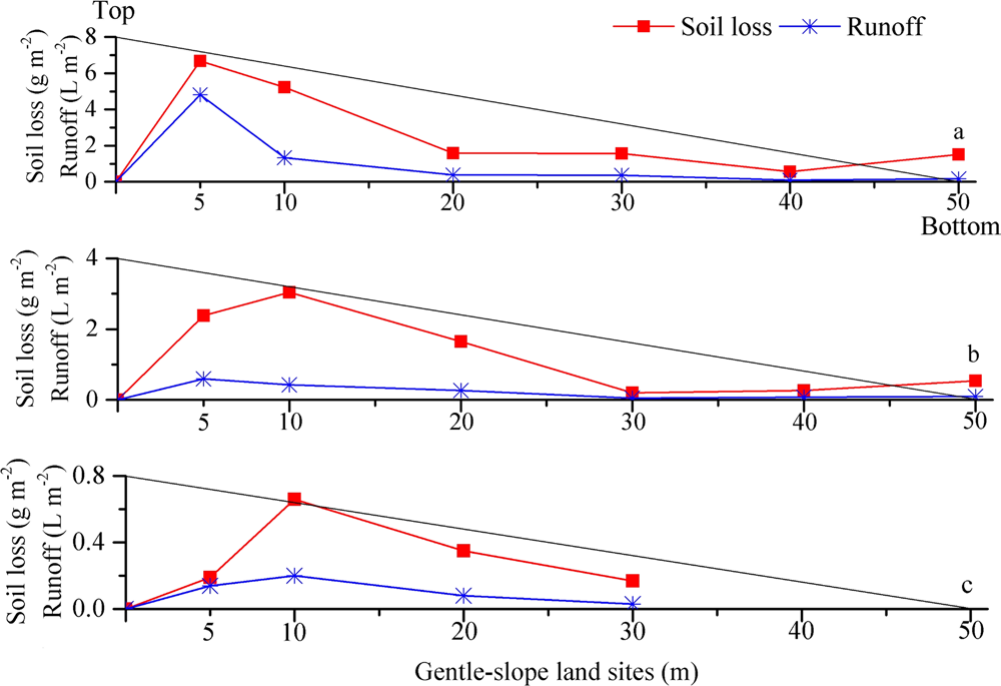
Soil loss and runoff at sites with different slope lengths (5, 10, 20, 30, 40 and 50 m) along gentle-slope lands under runoff scouring with diverse upslope inflow rates (**a**-30 L min^−1^; **b**-15 L min^−1^; and **c**-5 L min^−1^).

The amounts of soil loss and runoff at the sites with different slope lengths (5, 10, 20, 30, 40 and 50 m) along the gentle-slope lands under simulated overland flow with diverse upslope inflow rates are shown in Fig. 2. The ANOVA and T-test showed that the slope length had a significant effect on runoff and soil loss (*p* < 0.01). When the upslope inflow rate was 30 L min^−1^, the soil loss and runoff were largest at the 5 m gentle-slope land site. As the runoff flowed along the slope, it began to erode the soil and carry sediments; thus, the runoff required more energy to transport sediments on the slope. The energy required to erode the soil decreased to the minimum value at the 40 m gentle-slope land site (Fig. 2a). Under an upslope inflow rate of 15 L min^−1^, the soil loss and runoff initially increased and then decreased and finally increased. Both soil loss and runoff reached their maximum values at 10 m and then decreased to the 30 m gentle-slope land site; an increasing trend was noted at 30 m (Fig. 2b). Soil loss and runoff decreased with the decreasing upslope inflow rate, and no runoff and sediments were observed in 40 and 50 m gentle-slope land sites when the upslope inflow rate was 5 L min^−1^ (Fig. 2c). The trend of soil loss and runoff increased first and then decreased at the 10 m gentle-slope land site and reached a minimum at the 30 m gentle-slope land site (Fig. 2c).

**Figure 3:**
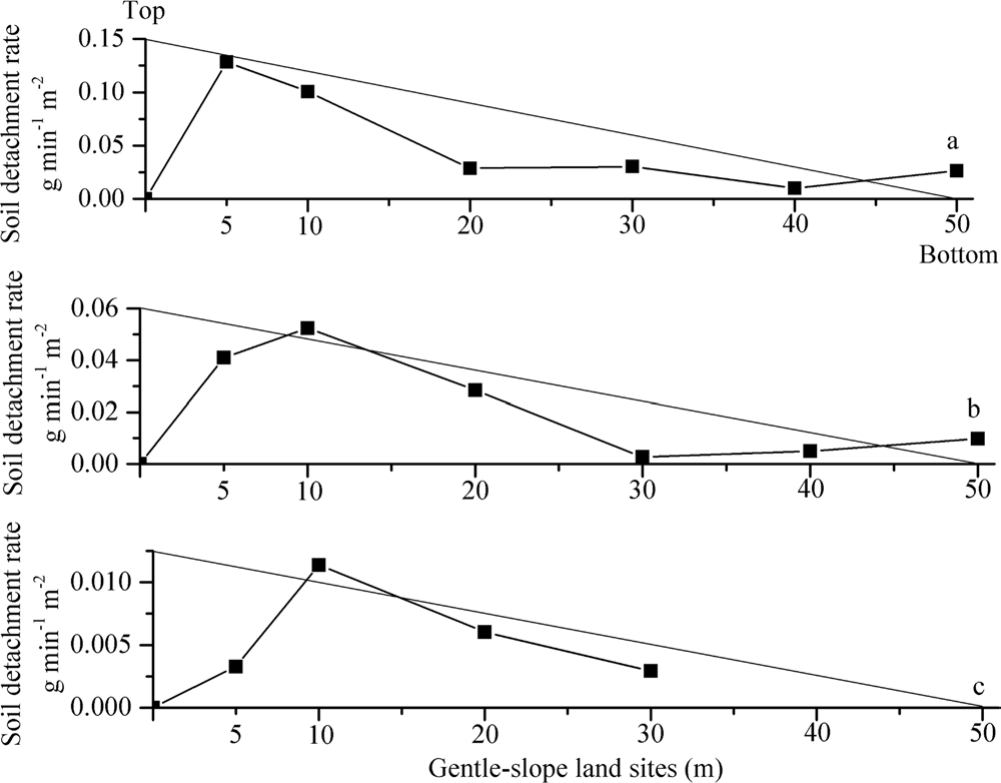
Soil detachment rates at sites with different slope lengths (5, 10, 20, 30, 40 and 50 m) along gentle-slope lands under runoff scouring with diverse upslope inflow rates (**a**-30 L min^−1^; **b**-15 L min^−1^; and **c**-5 L min^−1^).

**Figure 4:**
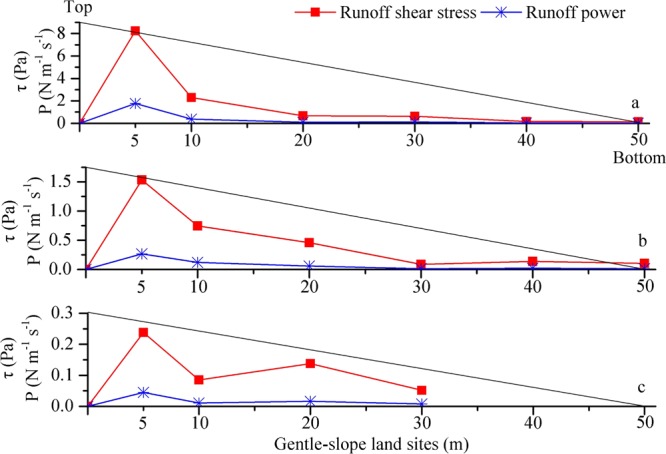
Runoff shear stress (*τ*) and runoff power (*Pa*) at sites with different slope lengths (5, 10, 20, 30, 40 and 50 m) along gentle-slope lands under runoff scouring with diverse upslope inflow rates (**a**-30 L min^−1^; **b**-15 L min^−1^; and **c**-5 L min^−1^).

To further account for the change tendency of soil loss and runoff at the different gentle-slope land sites, the soil detachment rate, runoff shear stress and runoff power were analyzed, as shown in Figs 3 and 4. The soil detachment rate was defined as the soil weight denuded per unit area and time^25,26^. The ANOVA and T-test showed that the slope length had a significant effect on the soil detachment rate, runoff shear stress and runoff power (*p* < 0.01) under different upslope inflow rates. When the upslope inflow rate was 30 L min^−1^, the runoff shear stress of the upslope inflow was too high (Fig. 4a) to produce a large soil detachment rate (Fig. 3a), which caused the occurrence and development of rapid scouring. However, the runoff sediment content was close to the sediment transport capacity of the flow as scouring persisted, causing the runoff shear stress and soil detachment to decrease. Although the soil detachment rate increased with the increasing slope length, there was little room for growth of the sediment transport capacity of the flow; thus, soil detachment presented a smaller change tendency. The soil detachment rate, runoff shear stress and runoff power of 15 and 5 L min^−1^ decreased gradually (Figs 3b,c and 4b,c).

**Figure 5:**
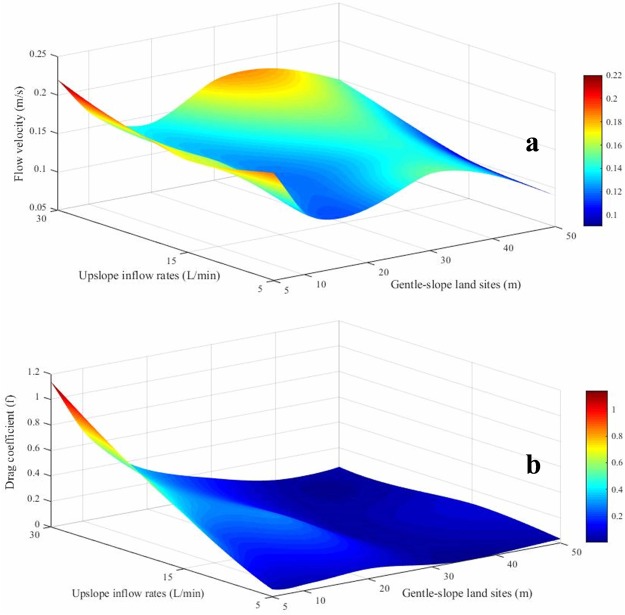
The flow velocity (*V*) and Darcy-Weisbach drag coefficient (*f*) at sites with different slope lengths along gentle-slope lands under runoff scouring with various upslope inflow rates.

The flow velocities at the sites with different slope lengths with diverse upslope inflow rates are shown in Fig. 5a. The flow velocity decreased and then increased with the increasing slope length, with a range from 0.10 to 0.22 m s^−1^. The fluctuation trend showed a change from strength to weakness with the increase in the slope length, and the amplitude of the velocity variation on slopes larger than 30 m was significantly weakened. Partial factor correlation analysis of the relationship between the upslope inflow rate, slope length and flow rate showed that the flow rate was significantly correlated with the upslope inflow rate (*r* = 0.592, *p* < 0.05) and slope length (*r* = −0.601, *p* < 0.05). This result showed that the slope length had a greater effect on the flow velocity of the slope than on the upslope inflow rate. The change trend of the drag coefficient shown in Fig. 5b ranged from 0.02 to 1.41. The *f* decreased significantly with the slope length with a slight upward trend at 30 m.

As shown in Table 2, soil loss was selected as the reference series and the parameters were selected as the comparison series. The relevancy between soil loss and correlation parameters was analyzed using the gray correlation method^27^. Each parameter had a correlation with the soil loss for newly reconstructed soil. The correlation order of the parameters to soil loss was ζ_V_ > ζ_Q_ > ζ_P_ > ζ_D_ > ζ_S_ > ζ_L_ > ζ_R_ > ζ_SS,_ and the range was 0.340–0.923. Specifically, the flow velocity had the largest influence on soil loss.

**Table 2:** Relevancy between soil loss and the correlation parameters.

Parameters	ζ_Q_	ζ_L_	ζ_R_	ζ_S_	ζ_SS_	ζ_P_	ζ_V_	ζ_D_
Relevancy	0.822	0.665	0.350	0.707	0.340	0.723	0.923	0.717

Note: ζ_Q_ represents the upslope inflow rate; ζ_L_ represents the slope length; ζ_R_ represents the runoff; ζ_S_ represents the soil detachment ratio; ζ_SS_ represents the runoff shear stress; ζ_P_ represents the runoff power; ζ_V_ represents the flow velocity; ζ_D_ represents the Darcy-Weisbach drag coefficient.

## Discussion

In hilly or mountainous regions of China, terracing is an important technique in land consolidation projects, and level terrace construction is preferred. However, constructing large horizontal terraced fields in the mountainous area of Chongqing is difficult. Therefore, gentle-slope lands are the most common type of cultivated field in the region. Land consolidation of farming plot construction engineering in hilly mountainous regions plays an important role in the mechanization, large-scale production and industrialization of agriculture. This approach also improves water and soil conservation, enhances soil fertility and expands areas that are accessible to agricultural machinery^5^. However, the positive effect of construction engineering requires years. The soil properties of reconstructed soil after farm plot construction with a loose structure and poor water holding capacity result in deterioration of its physical properties and cause soil erosion^20,28^. Soil erosion can cause loss of soil nutrients and a decline in soil fertility, which should receive adequate attention during terrace construction^17^. Liu *et al*.^8^ showed that soil erosion increased significantly at steep-sloped sites without protective measures during rainstorm events after land consolidation. The results of this study revealed that the farming plots of gentle-slope lands would be eroded after precipitation events or simulated overland flow. However, the degree of soil loss is also affected by the slope length, rainfall intensity and upslope inflow rate. Under natural conditions, the rainfall intensity cannot be controlled. Protective measures can be taken after construction, but such measures are expensive. The results of this study show that different degrees of soil erosion are produced by different slope lengths.

Slope length is one crucial factor affecting soil erosion^15,29^, and its influence is complex. Extensive research has been conducted on the relationship between slope length and runoff and sediment^11^. According to previous studies, there are three main viewpoints on the impact of slope length on soil erosion, (1) Soil erosion decreases with an increasing slope length^30,31^. (2) Erosion increases from uphill to downhill with the slope length^32^. (3) Soil erosion is a dynamic process with an increasing slope length^33^. On one hand, soil erosion is enhanced because the downhill water yield is greater than the uphill yield. On the other hand, energy is consumed to weaken erosion^34^. Therefore, these factories restrict each other. Kinnell^14^ showed that the effect of slope length on sediment discharge was highly dependent on variations in the runoff response resulting from variations in the rainfall duration-intensity-infiltration conditions rather than the plot length per se. The change in soil loss of newly reconstructed soil with different slope lengths in this study was complicated by “runoff degradation” during the unsteady period. Under natural rainfall and simulated overland flow conditions, providing continuous runoff is more difficult for a longer plot^35^. Therefore, the longer slope would reduce the loss of runoff downslope, resulting in more reinfiltration^36,37^. The “runoff degradation” phenomenon was observed on the 30 and 40 m slopes, which was similar to research showing that the runoff rate decreased with an increasing slope length^38^. One possible explanation is that a slope length of 30 or 40 m is the runoff continuity threshold. The catchment area of the slope was enlarged, the runoff pooling path was increased, the runoff was discontinuous and the infiltration intensity was enhanced, which led to a decrease in runoff. The gully cliff on both sides formed by water flow collapsed; thus, the water flow was blocked, resulting in an increase in the infiltration capacity and a decrease in runoff. The effects of slope length and the upslope inflow rate on the runoff rates were mainly due to the spatial variability of the downslope soil infiltration^39^ and reinfiltration^40^. At the same time, the soil loss characteristics varied among sites with different slope lengths; the degree of soil loss reached the minimum values on 30 or 40 m gentle-slope land sites under natural rainfall and simulated overland flow conditions. This phenomenon occurred because the sediment was constantly removed with the water flow with increasing slope length. Thus, the runoff required more energy to transport sediments on the slope^35^, and the energy used to entrain and disperse soil particles gradually weakened. With further extension of the slope length, the runoff energy was insufficient to transport more sediment when the sediment yield rate reached its maximum value. Because sediment began to deposit, the energy used to denude the soil increased and caused more erosion. In this way, the fluctuation of runoff and soil loss along the slope length indicated that soil erosion was a complex process of entrainment, transportation and deposition, and that these processes alternate and repeat. The entrainment of sediment particles was primarily dependent on the runoff shear stress under the water erosion cnditions^33^. The ability of water erosion to disperse soil particles increased with the increase in the upslope inflow, leading to an increase in the sediment concentration^41^. Meanwhile, the increase upslope inflow increased the runoff depth, causing the runoff shear stress and soil detachment rates to increase correspondingly^42^. The soil detachment rate decreased when the critical sediment discharge was reached^43^.

To promote large-scale development of agricultural mechanization, the length of the sloping terrace should be as long as possible. However, the cost of land consolidation and the degree of erosion should also be considered. The length should be within an appropriate range to optimize the conditions. The results of this study under erosive precipitation events or runoff scouring showed that the degree of soil erosion was lowest when the slope length was 30 or 40 m. Thus, 30–40 m may be the appropriate slope length range to control the loss of newly reconstructed soil from gentle-slope lands.

## Materials and Methods

### Study area

**Figure 6:**
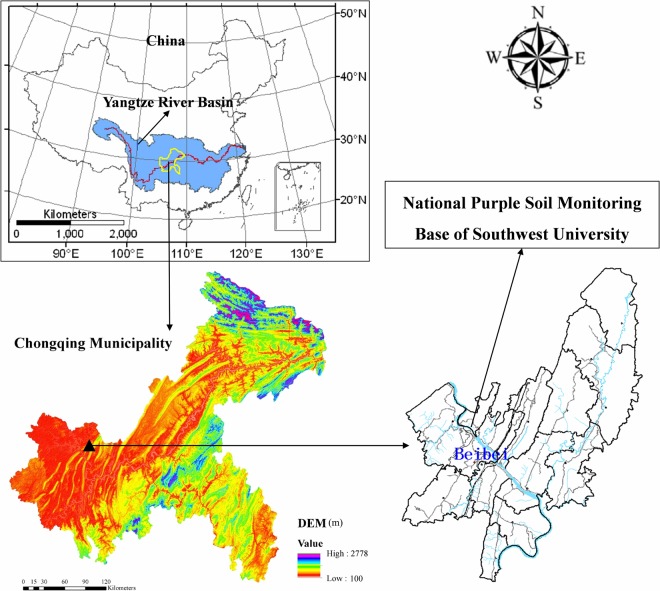
Location of the study area.

The study area was located in the National Purple Soil Monitoring Base of Southwest University, Beibei, Chongqing (106°26′E, 30°26′N) at an altitude of 230 m (Fig. 6). The area has a subtropical, humid climate with a mean annual temperature of 18.3 °C The average annual precipitation is 1105 mm, with 70% occurring between May and September. The average annual amount of sunshine is 1277 h, and the mean annual frost-free period is 334 d. The test soils, which are classified as Regosols in FAO Taxonomy or Entisols in USDA Taxonomy^44^, are formed from purple rocks and weathering products and mainly are distributed in the Sichuan Basin of southwestern China.

### Farming plot construction engineering

**Figure 7:**
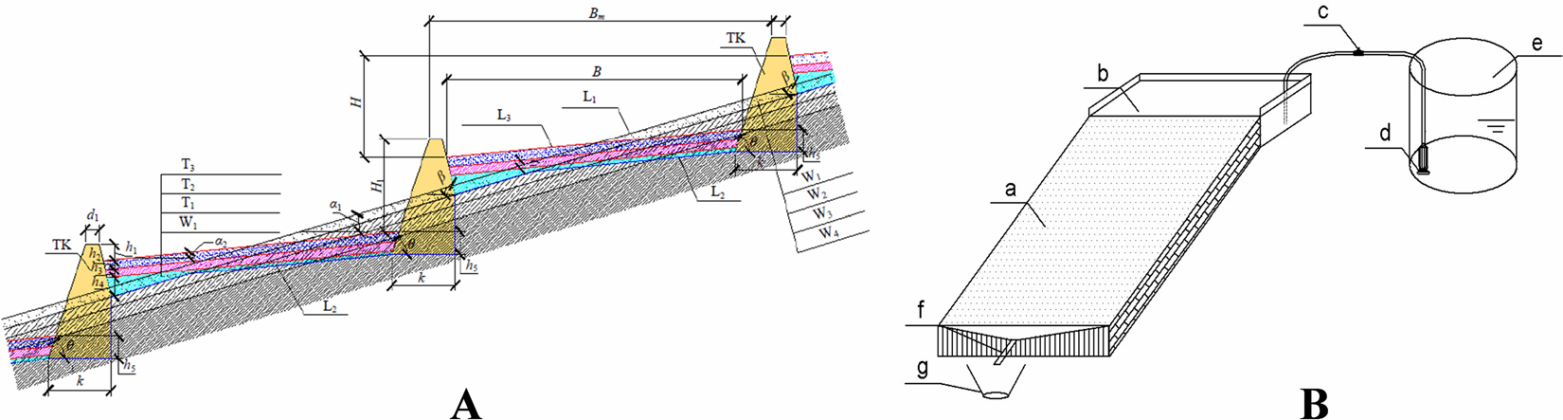
(**A**) A schematic diagram of gentle-slope land construction (Note: H: Height difference of the adjacent terrace, m; H_1_: Outside slope height of the ridge, m; d_1_: Top width of the ridge, m; k: Width of the ridge foundation, m. (**B**) Clear length of the terrace surface (excluding the ridge), m: B_m_: Length of the terrace surface (including the ridge), m; h_1_: Freeboard height of the ridge, m; h_2_: Depth of topsoil backfilling, m; h_3_: Depth of immature soil backfilling and compacting, m; h_4_: Depth of parent material and rock fragment backfilling, m; h_5_: Embedded depth of the ridge foundation, m; α_1_: Slope of the terrace before construction, 15°; α_2_:Slope of the terrace after construction, 10°; β: Inner slope of the ridge, (°); θ: Outer slope of the ridge, (°); TK: Ridge; L_1_: Ground line before construction; L_2_: Excavation line; L_3_: Ground line after construction; W_1_: Stripping topsoil; W_2_: Excavating and transporting the earthwork; W_3_: Excavating and transporting the parent material; W_4_: Blasting rock; T_1_: Backfilling parent material and rock fragment; T_2_: Backfilling and compacting immature soil; T_3_: Backfilling topsoil). (**B**) Layout of the experimental setup. (Note: a-Experimental plot; b-Overflow groove; c-Water value; d-Water pump; e-Water supply; f-Collecting groove; and g-Sample barrel).

First, the original 20-cm-thick topsoil was removed and deposited nearby. After engineering, the soil was reclaimed as topsoil for the farming plots. Then, the total soil thickness in the constructed plots was determined to be 50 cm. Finally, the deep excavation, refilling, land-reshaping, and leveling engineering measures were implemented. A schematic diagram of farming plot construction is shown in Fig. 7A.

**Figure 8:**
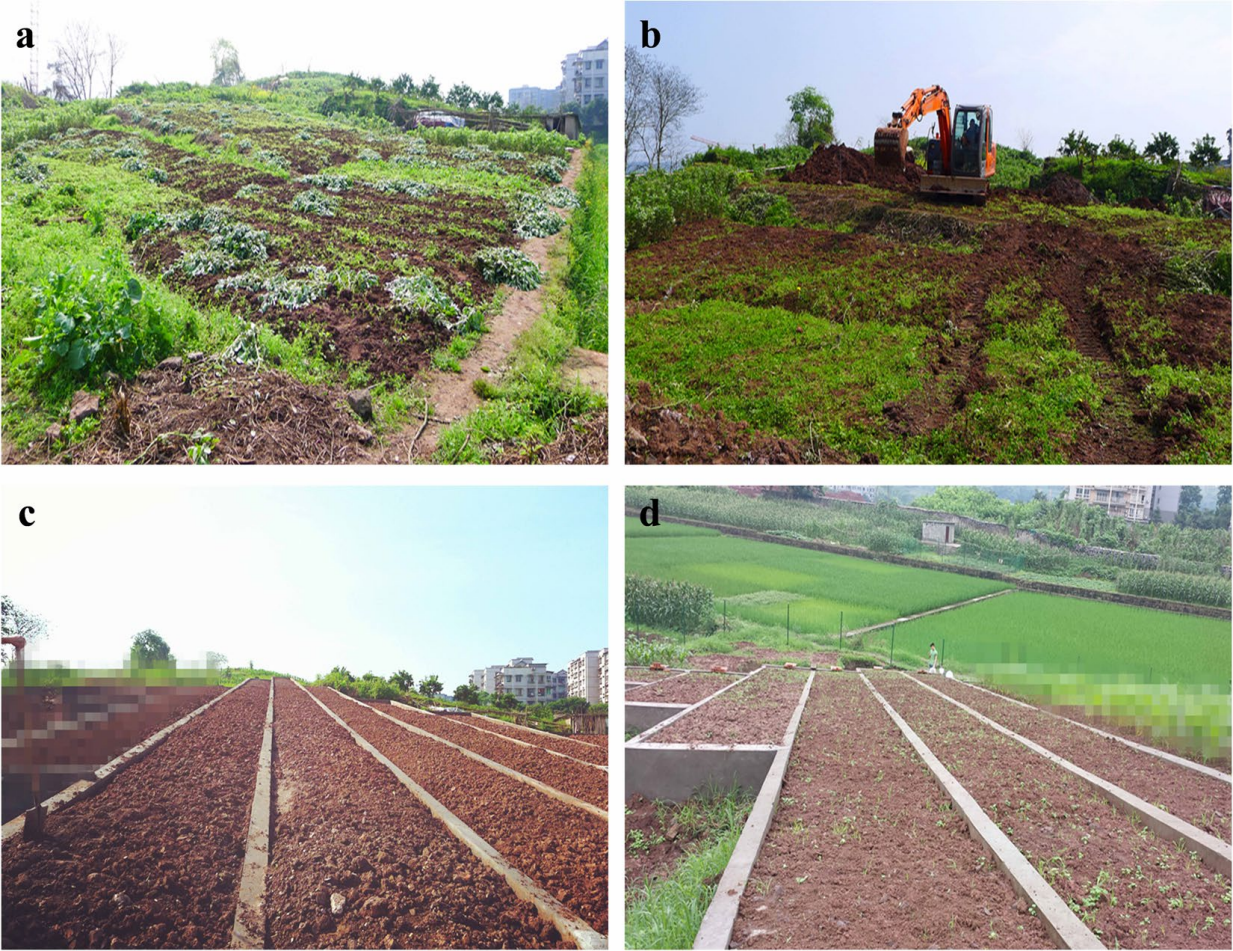
The experimental field plots before and after farming plot construction engineering.

To meet the requirements of mechanized farming, soil and water conservation, and cost effectiveness of rebuilding farmland, this study used a 10° slope gradient as a benchmark. A field plot with a slope gradient of 20°in the study area was selected and reduced to 10° by engineering. To clarify the effects of the slope lengths of the different sites on soil erosion and reduce the impact of sampling along the path, the experiment established six field plots with different slope lengths of 5, 10, 20, 30, 40 and 50 m, a field width of 2 m and a depth of 0.6 m. The bottom of the plot was the natural soil parent material separated by a cement ridge with a 0.2 m width. The cement ridge was built on the parent material layer, and a PVC sump was arranged at the end of the plot to collect runoff. Before the experiment, the topsoil (approximately 15 cm) was loosened, and the surface was raked to simulate general farming tillage. No fertilizer was applied to the experimental plots, and no crops were planted. The experimental field plots are shown before and after farming plot construction engineering in Fig. 8. The test soil was analyzed after engineering; the results are presented in Table 3.

**Table 3:** Properties of test soils from plots with different slope lengths.

Slope length (m)	Bulk density (g cm^−3^)	Initial moisture content (%)	Capillary porosity (%)	Soil particle composition			Organic content (g kg^−1^)	pH
				Sand (2–0.05 mm)	Silt (0.05–0.002 mm)	Clay (<0.002 mm)		
5	1.47 ± 0.12	16.82 ± 0.40	39.96 ± 0.57	28.39 ± 2.67	41.42 ± 2.41	30.18 ± 0.35	16.51 ± 0.40	5.59
10	1.46 ± 0.05	18.83 ± 0.04	37.71 ± 0.28	27.16 ± 2.44	43.05 ± 1.71	29.78 ± 3.43	16.04 ± 0.38	5.61
20	1.49 ± 0.06	17.86 ± 0.47	40.13 ± 0.71	31.89 ± 2.37	39.00 ± 0.87	29.11 ± 2.13	16.50 ± 0.35	5.62
30	1.48 ± 0.10	18.65 ± 0.37	40.24 ± 0.36	33.52 ± 5.03	37.80 ± 5.59	28.68 ± 0.93	16.05 ± 0.25	5.58
40	1.47 ± 0.15	16.83 ± 0.61	39.40 ± 0.40	36.19 ± 6.64	36.69 ± 3.01	27.12 ± 3.63	16.11 ± 0.26	5.60
50	1.48 ± 0.18	18.75 ± 0.44	38.96 ± 0.82	30.16 ± 4.69	39.85 ± 2.60	29.99 ± 2.38	16.20 ± 0.28	5.62

### Experimental design and methods

In addition to rainfall data from the rainy seasons, the simulated overland flow experiment was used to evaluate the soil loss process and the characteristics of the newly reconstructed soil on gentle-slope lands. The specific methods are described below.

### Natural rainfall

The rain gauge was set in an experimental plot and recorded precipitation after rainfall. Related data for each rainfall from the weather station of Beibei district were used as a reference. The linear distance of the experimental field from the weather station was 4.8 km. The runoff was quantitated in a runoff pond after rainfall, and the sediment was measured after runoff precipitation.

### Simulated overland flow

According to observation data concerning the local runoff and terrain conditions, the maximum runoff values used to determine the upslope inflow rates were 5, 15 and 30 L min^−1^; the rain intensity per unit area was 0.05–3 mm min^−1^. An overflow groove was placed at the upper end of the experimental plot, and the bottom of the overflow groove was covered with a few layers of gauze to prevent excessive erosion. The water storage barrel supplied water, and the overflow groove was located on top of the experiment plot horizontally to cause overland flow and maintain well-distributed sheet flow condition. A tap was equipped at the top of the overflow chute to regulate the flow discharge (Fig. 7B). Meanwhile, a voltage regulation pump and a flow meter were used to provide stable flow discharge with the error controlled to within 5%. To avoid the effects of surface roughness and the initial soil moisture content on the test results, the test plots were raked and shelved for approximately one week before initiation of each experiment. Before the experiment, the test soil was placed 12 h after saturation, and the physical properties of each plot, such as the soil bulk density, initial water content and mechanical composition, were measured on the upper, middle and lower sections of the slope surface. To collect surface runoff after runoff generation, approximately 550 mL of runoff and sediment was collected at 1 min in the first 10 min and then collected at 3 min after the first 10 min. The flow velocity of the slope surface was measured according to the potassium permanganate staining method^45^ when the runoff was stable, and the average value was determined several times. Because the velocity of the runoff measured using the potassium permanganate staining method was the dominant flow velocity of the slope, the measured velocity was multiplied by the correction factor 0.75 as the average velocity of the cross section of the water flow^46^. The experimental time period was during September-October, 2017.

### Equations and data treatment

The soil detachment rate (*D*_*r*_, g min^−1^ m^2^) was calculated with the following equation:

1 $${D}_{r}=\frac{M}{B\cdot L\cdot T}$$

where *M* is the mass of sediment collected during the observation time T(g), *B* is the width of the water-crossing section (m) *B* = 2 m, *L* is the slope length (m); and *T* is the observation time (min).

Runoff shear stress (*τ*, Pa) was calculated by the following equation:

2 $$\tau =\rho \cdot g\cdot R\cdot J$$

where *ρ* is the water density (kg m^−3^), g is the gravitational acceleration (m s^−2^), *J* (m/m) is the sine value of the slope gradient sin 10° = 0.174 and *R* is the hydraulic radius, which was considered equal to the mean flow depth (*H*) under the overland flow condition (m), *H* can be estimated by

3 $$H=\frac{{R}_{0}}{V\cdot B\cdot T}$$

where *R*_0_ is the runoff during the observation time *T* (m^3^), and *V* is the mean calculated flow velocity (m s^−1^).

Runoff power (*P*, N m^−1^ s^−1^) was calculated by the following equation:

4 $$P=\tau \cdot V$$

The Darcy-Weisbach drag coefficient (*f*) was calculated by the following equation:

5 $$f=\frac{8R\cdot J\cdot g}{{V}^{2}}$$

## Conclusions

Based on natural rainfall and simulated overland flow, this study investigated the soil erosion characteristics of newly reconstructed gentle-slope lands with different slope lengths (5, 10, 20, 30, 40 and 50 m). The results indicated that the degree of soil erosion under erosive precipitation events or simulated overland flow was lowest for a slope length of 30 or 40 m. The runoff sediment content was close to the sediment transport capacity of the flow as scouring persisted, causing the runoff shear stress and soil detachment to decrease. The soil detachment rate, runoff shear stress and runoff power exhibited the same trends as the soil and runoff loss. Thus, 30–40 m appears to be the appropriate slope length range to control soil loss from newly reconstructed soil. The results and recommendations reported herein will have value for newly reconstructed soil in farming plot construction engineering in hilly mountainous regions. In the future, the sediment sorting and transport mechanism and the mobilization of nutrients from newly reconstructed soil deserve further in-depth study.

## Data Availability

The original data can be obtained from the authors upon reasonable request.
